# Patient and anesthesia characteristics of children with low pre‐incision blood pressure: A retrospective observational study

**DOI:** 10.1111/aas.13520

**Published:** 2019-12-22

**Authors:** Wietze Pasma, Linda M. Peelen, Stefanie van den Broek, Stef van Buuren, Wilton A. van Klei, Jurgen C. de Graaff

**Affiliations:** ^1^ Department of Anesthesiology University Medical Center Utrecht Utrecht University Utrecht the Netherlands; ^2^ Department of Epidemiology Julius Center for Health Sciences and Primary Care Utrecht University Utrecht the Netherlands; ^3^ Department of Methodology & Statistics, FSS University of Utrecht Utrecht the Netherlands; ^4^ Netherlands Organization for Applied Scientific Research TNO Delft the Netherlands; ^5^ Department of Anesthesiology Erasmus MC—Sophia Children's Hospital Rotterdam the Netherlands

## Abstract

**Background:**

Intraoperative blood pressure has been suggested as a key factor for safe pediatric anesthesia. However, there is not much insight into factors that discriminate between children with low and normal pre‐incision blood pressure. Our aim was to explore whether children who have a low blood pressure during anesthesia are different than those with normal blood pressure. The focus of the present study was on the pre‐incision period.

**Methods:**

This retrospective study included pediatric patients undergoing anesthesia for non‐cardiac surgery at a tertiary pediatric university hospital, between 2012 and 2016. We analyzed the association between pre‐incision blood pressure and patient‐ and anesthesia characteristics, comparing low with normal pre‐incision blood pressure. This association was further explored with a multivariable linear regression.

**Results:**

In total, 20 962 anesthetic cases were included. Pre‐incision blood pressure was associated with age (beta −0.04 SD per year), gender (female −0.11), previous surgery (−0.15), preoperative blood pressure (+0.01 per mm Hg), epilepsy (0.12), bronchial hyperactivity (−0.18), emergency surgery (0.10), loco‐regional technique (−0.48), artificial airway device (supraglottic airway device instead of tube 0.07), and sevoflurane concentration (0.03 per sevoflurane %).

**Conclusions:**

Children with low pre‐incision blood pressure do not differ on clinically relevant factors from children with normal blood pressure. Although the present explorative study shows that pre‐incision blood pressure is partly dependent on patient characteristics and partly dependent on anesthetic technique, other unmeasured variables might play a more important role.

**Editorial Comment**

Low blood pressure in anesthetized children before the surgical incision is not desirable. This retrospective study tried to find associated factors in a large pediatric surgical cohort. The findings from this analysis suggest that pre‐incision low blood pressure occurs in children with different characteristics and exposure factors. Concurrent regional anesthesia and intubation was the factor most strongly associated with low pre‐incision blood pressure.

## 1 INTRODUCTION

During anesthesia, vital signs such as blood pressure are monitored according to standards and guidelines.^1^ In 2016, reference curves for age‐appropriate blood pressure measurements under anesthesia were developed, using data from over 100 000 children across 11 centers.^2^ These curves show the relation between age, weight, or height and blood pressure during anesthesia, and allow us to compare these with actual blood pressure measurements during surgical care. The references were developed for a relatively stable period, most likely not influenced by anesthetic and surgical factors (eg, post‐induction dip in blood pressure and stress reaction on incision). In the next step, it has to be elucidated which children fall below these references or, in other words, which patients are at outliers of the reference values?

In adults, there is evidence that older age, higher ASA‐physical status, and co‐existing conditions such as hypertension, diabetes mellitus, and myocardial infarction are associated with intraoperative hypotension. Additionally, in adults, researchers reported that intraoperative low blood pressure is associated with organ injury and adverse outcomes such as prolonged postoperative stay and death. On the other hand, in the pediatric population, this evidence is not present, although blood pressure has been suggested as a key factor for safe pediatric anesthesia.^3^, ^4^, ^5^, ^6^, ^7^, ^8^ In contrast to research in adults, only a few studies are available in children, where fasting status, ASA physical status, preoperative hypotension, intravenous induction, propofol dosage, and body mass index were found to be associated with intraoperative hypotension in children.^9^, ^10^, ^11^

The aim of this study was to explore whether children who have a low blood pressure during anesthesia are different than those with normal blood pressure. The focus of the present study was on the pre‐incision period. We hypothesized that several pre‐existing patient characteristics are associated with pre‐incision blood pressure and that differences in the management of anesthesia induction, such as differences in medication dosage are associated with pre‐incision blood pressure.

## 2 METHODS

This retrospective cohort study included all non‐cardiac pediatric anesthetic procedures performed at a specialized tertiary referral university hospital (Wilhelmina Children's Hospital Utrecht, the Netherlands), from January 1, 2012 until December 31, 2016. Similar to the previous study in which the reference curves were developed,^2^ we excluded cardiac procedures or when the surgical specialty was missing. Also, at least two blood pressure measurements had to be available before incision. If the time of incision was not available, the procedure was excluded. All data were retrospectively collected from the Anesthesia Information Management System (AIMS, Anstat, version 2.0.4, 2015, Carepoint) and Electronic Health Record (HiX, Chipsoft). The IRB waived the need for informed consent under the Dutch Data Protection Act (METC number 16/235). We de‐identified the data before analysis.

## 3 BLOOD PRESSURE

We based low pre‐incision blood pressure on non‐invasive mean arterial blood pressure measurements, as this parameter, rather than systolic or diastolic blood pressure, is the key parameter in the local protocol for intraoperative blood pressure control. Non‐invasive blood pressure is measured according to protocol at least every 5 minutes by oscillometry and stored in the AIMS database. For our definition of low pre‐incision blood pressure, we collected measurements within 20 minutes before the start of the procedure (marked by an event registration of start incision) and calculated the mean of the last three of these measurements, which was the same method as was used in the development of the previously published references. Before the collection of measurements, we removed measurements that were defined as artifacts, that is, when the diastolic pressure was lower than 3 mm Hg, when the systolic pressure was equal or higher than 250 mm Hg, when the pulse pressure (systolic pressure minus diastolic pressure) was lower than or equal to 5 mm Hg or when one of the systolic, diastolic, or mean arterial pressure values was missing.^2^ Subsequently, using the reference curves, we calculated a standardized pre‐incision blood pressure (Z‐score) given the patient's height and gender using the relevant reference curve for mean arterial blood pressure values in the pre‐incision period.^2^ We collected height values within a clinically relevant time period before surgery, whereby this period depended on patient age (see Data S1). If no height measurement was available within this period, we considered height as missing data. Finally, we defined low pre‐incision blood pressure as a standardized blood pressure value (Z) lower than −2 standard deviations (SD) (ranging from 19‐48 mm Hg, for height 45‐200 cm). We considered a standardized blood pressure between −2 SD and 2 SD (55‐105 mm Hg, for height 45‐200 cm) as normal, and standardized blood pressure above 2 SD as high.^2^ We purposefully do not define hypotension in this study, which would imply that the blood pressure below a threshold is too low and harmful. Since there is no clear consensus on a hypotension definition for pre‐incision blood pressure in children under anesthesia, the choice of cut‐off value in this paper was arbitrary.

## 4 PATIENT AND ANESTHESIA CHARACTERISTICS

Characteristics were divided into patient and anesthesia‐related characteristics. Patient characteristics collected for this study were gender, age, preoperative height, preoperative weight, ASA physical status, preoperative blood pressure (in mm Hg), time of the start of the procedure (morning: 8 am to 12 am; afternoon: 12 am to 5 pm; evening till midnight: 5 pm to 12 pm; and after midnight: 12 pm to 8 am), previous surgery, and pre‐operative comorbidities. These factors have been studied before and were associated with blood pressure in children or in adults.^3^, ^9^, ^10^

We collected comorbidity data from preoperative evaluation charts, where we focused on comorbidities we assumed likely related to intraoperative blood pressure. When preoperative evaluation was performed but information on comorbidities was (partly) missing, we assumed that these comorbidities were not present. Procedure characteristics which we considered were as follows: surgical specialty, priority status, anesthetic technique performed, medication use during pre‐incision phase (propofol, atracurium, and sufentanil), and the inspired sevoflurane concentration.^3^, ^10^, ^12^, ^13^, ^14^, ^15^ The start of the pre‐incision period during which medication data were collected, was defined by the first of the three bloodpressure measurements that were used to calculate the pre‐incision blood pressure. The end of this period was equal to the start of the surgical procedure. From this period, the median of inspired sevoflurane concentration was used.

## 5 STATISTICAL ANALYSES

In the first part of the analyses, we described the characteristics of the patients and the procedures in which a low pre‐incision blood pressure occurred and compared these characteristics to children with a normal pre‐incision blood pressure. Hence, we excluded cases with a relatively high blood pressure (>2 SD) for this part of the analysis. For continuous data, the median and interquartile range are presented. Because of the large sample size, we assumed the variance to be normally distributed, and compared the groups using a *t* test. In case of categorical and dichotomous variables, the data are presented as counts and percentages and groups are compared with a chi‐squared test.

In the second part of the analyses, we assessed the association between patient and anesthesia characteristics versus blood pressure using the calculated Z‐scores as a continuous outcome variable using multivariable linear regression analysis. Cases with a high blood pressure (Z > 2SD) were included in these analyses. We included the same patient characteristics—excluding height, weight, and ASA physical status because of expected collinearity—and anesthesia characteristics into a linear regression model with standardized pre‐incision blood pressure as outcome. This first and second part of the analyses were defined before prior to obtaining the data. The study was designed and reported according to the STROBE guidelines.

As a post‐hoc analysis, to investigate whether potential risk factors were different for infants and older children, we fitted the same model in children younger than 12 months and children older than 12 months separately.

Missing data rarely occur completely at random and conducting complete case analysis typically leads to biased effect estimates.^16^, ^17^ Therefore, we used multiple imputation using the *mice* package.^18^ We imputed 20 complete datasets, in which we used passive imputation to impute Z‐values for pre‐incision blood pressure. We pooled effect estimates and test statistics in individual imputation sets using Rubin's rules.^16^ Results presented throughout the manuscript are based on these imputed data.

We extracted and processed the data from our local enterprise data warehouse, using SAS software (Version 9.4, Copyright © 2013 SAS Institute Inc). We further processed and analyzed the de‐identified data in R (R Foundation for Statistical Computing. https://www.R-project.org, version 3.3.2 [2016‐10‐31]). We considered a *P* value < .05 statistically significant throughout the analyses.

## 6 RESULTS

### 6.1 Cohort selection

**Figure 1:**
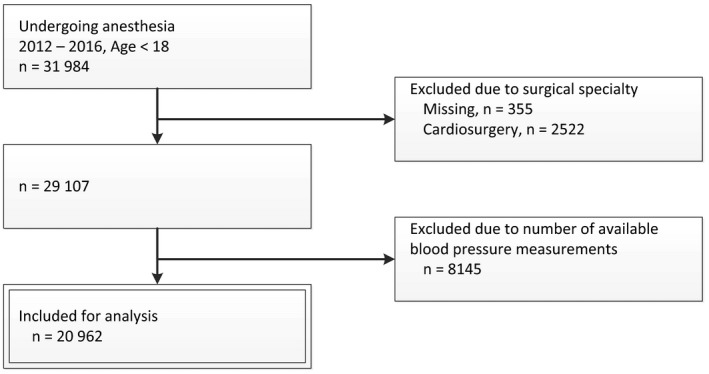
Flowchart of included anesthesia procedures for analysis. Double framed boxes indicate the included procedures that were used for analysis. 20 962 procedures were included for the multivariable regression analyses. *Measurements of height were considered expired, when it was not measured within a clinical relevant time before surgery (see Data S1)

Within the study period, we identified 31 984 pediatric anesthesia procedures. We excluded 2 877 (9%) cases as these were cardiac procedures or the surgical specialty was missing. An additional 8 145 (25%) cases were excluded because the number of measurements was too low (less than 2). This resulted in 20 962 included procedures (Figure 1). Distribution of gender, age, ASA physical status, and surgical specialty of all procedures and those included in our analysis are provided in Table 1. We selected three blood pressure measurements within 20 minutes before incision, which we used to calculate the standardized blood pressure. The median period in which these measurements were selected, was 10 minutes (IQR 7‐12). The within patient variation of these blood pressure measurements was low. The median of the standard deviation per patient was 4.2 mm Hg (IQR 2.1‐7.8 mm Hg) and the median of the range (maximum minus minimum) was 8 (IQR 4‐14).

**Table 1:** Baseline characteristics of included anesthetic procedures (n = 20 962)

Parameter	n	(%)
Number of cases	20 962	
Age		
Neonates (0‐1 mo)	516	(2.5%)
Infant (1 mo‐1 y)	3342	(15.9%)
1‐4 y	4776	(22.8%)
4‐8 y	4132	(19.7%)
8‐18 y	8196	(39.1%)
Gender		
Male	8440	(40.3%)
Female	12 522	(59.7%)
ASA physical status		
1	10 213	(48.7%)
2	6644	(31.7%)
>2	1541	(7.4%)
Unknown	2564	(12.2%)
Surgical specialty		
Pediatric surgery	4509	(21.5%)
Maxillofacial	828	(4.0%)
Neurosurgery	872	(4.2%)
Ophthalmology	1549	(7.4%)
Otolaryngologic surgery	3461	(16.5%)
Pediatric intervention	3916	(18.7%)
Reconstructive surgery	1473	(7.0%)
Urologic surgery	4354	(20.8%)

Note

Categorical data are presented as number of procedures and percentage.

### 6.2 Low versus normal blood pressure

In total, 6.1% (n = 1 259) of the procedures patients had a low pre‐incision blood pressure. For this part of the analysis, 477 cases with high pre‐incision blood pressure were excluded. The comparison of patient and anesthesia characteristics showed that children with low blood pressure were older, more often female, weighed more, were longer in height, had a higher pre‐operative blood pressure, had more often previous surgery, were more often operated after office hours (after 5 pm), had more often movement disorders, and had more often kidney disorders (Table 2). In addition, these children underwent procedures of different surgical disciplines. Emergency surgery was less common in children with a low pre‐incision blood pressure. The combination of general and a loco‐regional anesthesia and a tube (instead of a supraglottic airway) was more often used in the low blood pressure group. Dosage of pre‐incision medication was similar in both groups, and concentration of sevoflurane was lower in the low blood pressure group (Table 3).

**Table 2:** Comparison of patient characteristics between anesthesia procedures with low and normal pre‐incision blood pressure

Parameter	Low blood pressure		Normal blood pressure		*P* value
Group size	1259		19 226		
Age (years)	8.1	(4.8‐12.7)	5.5	(1.4‐11.3)	**
Patient gender					
Male	716	(56.9%)	11 533	(60%)	*
Patient weight (kg)	26	(17.6‐43)	19.6	(10.7‐37)	**
Patient height (cm)	129	(105‐153)	110	(78.3‐145)	**
ASA physical status					
1	731	(58.1%)	10 583	(55%)	
2	413	(32.8%)	6928	(36%)	
>2	115	(9.1%)	1715	(8.9%)	
Pre‐operative NIBP	77	(71‐84)	78	(72‐85)	
Time of surgery					
After midnight	15	(1.2%)	202	(1.1%)	*
Morning	389	(30.9%)	6329	(32.9%)	
Afternoon	814	(64.6%)	11 725	(61.0%)	
Evening till midnight	42	(3.3%)	970	(5.0%)	
Patient had previous surgery	917	(72.9%)	12 097	(62.9%)	**
Bleeding disorders	24	(1.9%)	424	(2.2%)	
Cardiac history	134	(10.7%)	1734	(9%)	
Coagulation disorders	29	(2.3%)	499	(2.6%)	
Bronchial hyperreactivity	18	(1.4%)	267	(1.4%)	
Movement disorder	158	(12.5%)	1871	(9.7%)	*
Apnea	23	(1.8%)	274	(1.4%)	
Epilepsy	45	(3.6%)	831	(4.3%)	
Kidney disorders	106	(8.4%)	1279	(6.7%)	*
Liver disorders	16	(1.3%)	218	(1.1%)	
Lung disorders	138	(11.0%)	1958	(10.2%)	
Recurrent airway disorders	21	(1.7%)	386	(2.0%)	

Note

Low blood pressure is defined as values below −2SD and normal blood pressure as values between −2SD and +2SD. Pre‐incision reference values, corrected for height and gender were used. Due to pooling and rounding of results, these numbers might not add up to group totals. Continuous data are presented as median and interquartile range, categorical data are presented as number of procedures and percentage.

* *P* value < .05.

** *P* value < .001.

**Table 3:** Comparison of procedure characteristics between anesthesia procedures with low and normal pre‐incision blood pressure

Parameter	Low blood pressure		Normal blood pressure		*P* value
Group size	1259		19 226		
Surgical discipline					
Pediatric surgery	308	(24.5%)	4143	(21.5%)	**
Maxillofacial	52	(4.2%)	772	(4.0%)	
Neurosurgery	40	(3.2%)	814	(4.2%)	
Ophthalmology	61	(4.8%)	1462	(7.6%)	
Otolaryngologic surgery	274	(21.8%)	3081	(16.0%)	
Pediatric intervention	110	(8.7%)	3579	(18.6%)	
Reconstructive surgery	94	(7.5%)	1371	(7.1%)	
Urologic surgery	319	(25.3%)	4005	(20.8%)	
Priority status of surgery					
Emergency	159	(12.6%)	3084	(16.0%)	*
Planned	1100	(87.4%)	16 142	(84.0%)	
Loco‐regional technique used	486	(38.6%)	4946	(25.7%)	**
Artificial airway used					
Supraglottic airway	577	(45.8%)	9682	(50.4%)	*
Tube	682	(54.2%)	9544	(49.6%)	
Inspired sevoflurane (%)	2.7	(2.1‐3.4)	3.0	(2.3‐3.8)	**
Propofol (mg/kg)^a^	0.0	(0.0‐3.1)	0.0	(0.0‐2.9)	
Sufentanil (mg/kg)^a^	0.1	(0.0‐0.1)	0.1	(0.0‐0.1)	*
Atracurium (mg/kg)^a^	0.0	(0.0‐0.4)	0.0	(0.0‐0.4)	*

Note

Low blood pressure is defined as values below −2SD and normal blood pressure as values between −2SD and +2SD. Pre‐incision reference values, corrected for height and gender were used. Due to pooling and rounding of results, these numbers might not add up to group totals.

a If this anesthetic medication was not given, dose of zero mg/kg is included in the analysis. Continuous data are presented as median and interquartile range, categorical data are presented as number of procedures and percentage.

* *P* value < .05.

** *P* value < .001.

### 6.3 Characteristics versus blood pressure

**Figure 2:**
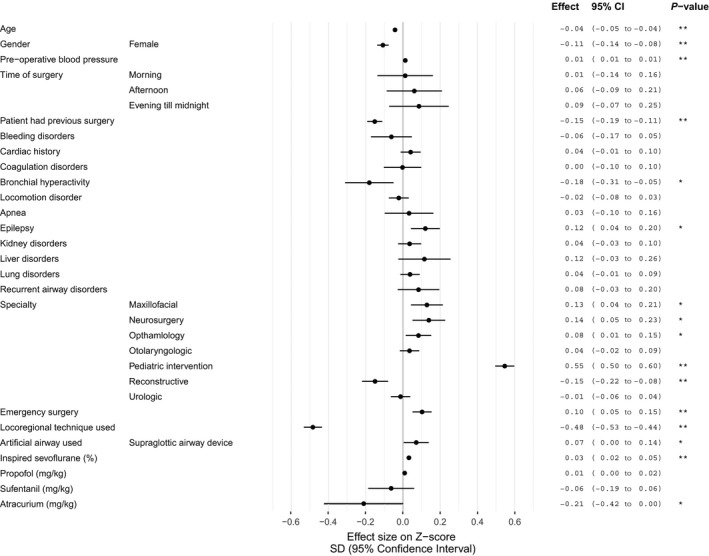
Forest plot of results of linear regression model for association between patient and procedure characteristics vs standardized pre‐incision blood pressure (Z‐score). Effect sizes are in Z‐score (unit is 1 standard deviation (SD)) with a 95% confidence interval (95% CI) of pre‐incision blood pressure. For time of surgery, the reference was midnight till morning, for specialty the reference was pediatric surgery and for artificial airway the reference was tube. *P value < .05, **P value < .001

Figure 2 presents the results from the multivariable regression analyses using the continuous Z‐values as an outcome and both patient and anesthesia characteristics as determinants. Older children (beta −0.04 SD per year [95% confidence interval−0.05 to −0.04]) and females (−0.11 [−0.14 to −0.08]) had a lower pre‐incision blood pressure, as did children who underwent surgery previously (−0.15 [−0.19 to −0.11]) or those with a lower preoperative blood pressure (0.01 [0.01‐0.01]). Children with epilepsy (0.12 [0.04‐0.20]) had a higher pre‐incision blood pressure and bronchial hyperreactivity (−0.18 [−0.31 to −0.05]) was associated with a lower pre‐incision blood pressure.

The use of a loco‐regional technique additionally to general anesthesia (−0.48 [−0.53 to −0.44]) was associated with a lower pre‐incision blood pressure. The use of supraglottic airway device (laryngeal mask airway) (0.07 [0.00‐0.14]) was associated with a higher pre‐incision blood pressure, compared to the use of an endotracheal tube. The dosage of propofol, atracurium, and sufentanil was not associated with pre‐incision blood pressure. In contrast, a higher concentration of sevoflurane (0.03 per sevoflurane % [0.02‐0.05]) was associated with an increase in pre‐incision blood pressure.

### 6.4 Different age groups

As a post‐hoc analysis, the model was fitted in infants and older children separately. The model for children under 12 months (Table 4) showed fewer significant associations than the model with the older children (Table 5). Age, pre‐operative blood pressure, surgical specialty, and use of loco‐regional technique are associated with pre‐incision blood pressure in both groups. Gender, previous surgery, epilepsy, inspired sevoflurane, and sufentanil dosage are associated with pre‐incision blood pressure in children over 12 months, but not in infants, whereas lung disorders and choice of artificial airway are associated with pre‐incision blood pressure in infants, but not in older children.

**Table 4:** Multivariable linear regression analysis of factors associated with standardized pre‐incision blood pressure in infants, defined as children younger than 12 months of age (n = 3858)

Parameter	Effect estimate (95% CI)		*P* value
Age (years)	0.25	(0.11‐0.38)	**
Female	−0.03	(−0.11 to 0.04)	
Pre‐operative blood pressure	0.01	(0.01‐0.02)	**
Time of surgery			
Midnight till morning (reference)			
Morning	−0.13	(−0.45 to 0.18)	
Afternoon	−0.18	(−0.49 to 0.13)	
Evening till midnight	−0.07	(−0.41 to 0.26)	
Patient had previous surgery	0.02	(−0.07 to 0.11)	
Bleeding disorders	0.19	(−0.33 to 0.71)	
Cardiac history	0.08	(−0.04 to 0.20)	
Coagulation disorders	−0.05	(−0.39 to 0.29)	
Bronchial hyperreactivity	−0.02	(−0.39 to 0.35)	
Locomotion disorder	−0.02	(−0.25 to 0.21)	
Apnea	0.09	(−0.32 to 0.49)	
Epilepsy	−0.03	(−0.32 to 0.26)	
Kidney disorders	0.10	(−0.02 to 0.22)	
Liver disorders	0.00	(−0.35 to 0.34)	
Lung disorders	0.19	(0.05‐0.32)	*
Recurrent airway disorders	−0.09	(−0.33 to 0.14)	
Surgical specialty			
Pediatric surgery (reference)			
Maxillofacial	−0.45	(−1.15 to 0.26)	
Neurosurgery	0.00	(−0.17 to 0.16)	
Opthalmology	−0.10	(−0.33 to 0.14)	
Otolaryngologic surgery	0.43	(0.29 to 0.57)	**
Pediatric intervention	0.39	(0.26 to 0.51)	**
Reconstructive surgery	−0.11	(−0.25 to 0.03)	
Urologic surgery	0.10	(−0.01 to 0.20)	
Emergency surgery	0.04	(−0.06 to 0.13)	
Loco‐regional technique used	−0.28	(−0.38 to −0.19)	**
Artificial airway used			
Tube (reference)			
Supraglottic airway device	0.21	(0.12‐0.31)	**
Inspired sevoflurane (%)	0.02	(0.00‐0.05)	
Propofol (mg/kg)	−0.02	(−0.04 to 0.00)	
Sufentanil (mg/kg)	0.18	(−0.02 to 0.37)	
Atracurium (mg/kg)	−0.08	(−0.23 to 0.07)	

Note

Effect sizes are presented as betas and should be interpreted as follows: an increase in one unit of the covariate will increase the blood pressure Z‐value (standardized pre‐incision non‐invasive blood pressure) by beta times the SD and 95% confidence intervals (CI).

* *P* value < .05.

** *P* value <0.001.

**Table 5:** Multivariable linear regression analysis of factors associated with standardized pre‐incision blood pressure in children older than 12 months of age (n = 17 104)

Parameter	Effect estimate (95% CI)		*P* value
Age (years)	−0.02	(−0.03 to −0.02)	**
Female	−0.10	(−0.13 to −0.06)	**
Pre‐operative blood pressure	0.01	(0.01‐0.02)	**
Time of surgery			
Midnight till morning (reference)			
Morning	0.04	(−0.12 to 0.20)	
Afternoon	0.11	(−0.06 to 0.27)	
Evening till midnight	0.15	(−0.03 to 0.32)	
Patient had previous surgery	−0.08	(−0.13 to −0.04)	**
Bleeding disorders	−0.05	(−0.16 to 0.06)	
Cardiac history	0.01	(−0.05 to 0.07)	
Coagulation disorders	0.01	(−0.09 to 0.11)	
Bronchial hyperreactivity	−0.13	(−0.28 to 0.01)	
Locomotion disorder	0.00	(−0.05 to 0.05)	
Apnea	0.10	(−0.04 to 0.23)	
Epilepsy	0.16	(0.08 to 0.24)	**
Kidney disorders	−0.06	(−0.13 to 0.01)	
Liver disorders	0.13	(−0.02 to 0.29)	
Lung disorders	0.01	(−0.04 to 0.07)	
Recurrent airway disorders	0.09	(−0.04 to 0.21)	
Surgical specialty			
Pediatric surgery (reference)			
Maxillofacial	0.21	(0.12‐0.30)	**
Neurosurgery	0.27	(0.17‐0.38)	**
Opthalmology	0.21	(0.14‐0.28)	**
Otolaryngologic surgery	0.10	(0.04‐0.15)	*
Pediatric intervention	0.65	(0.60‐0.71)	**
Reconstructive surgery	−0.08	(−0.16 to 0.00)	*
Urologic surgery	0.11	(0.05‐0.18)	**
Emergency surgery	0.06	(0.00‐0.12)	*
Loco‐regional technique used	−0.61	(−0.67 to −0.55)	**
Artificial airway used			
Tube (reference)			
Supraglottic airway device	0.07	(−0.03 to 0.17)	
Inspired sevoflurane (%)	0.04	(0.03‐0.06)	**
Propofol (mg/kg)	0.02	(0.00‐0.04)	*
Sufentanil (mg/kg)	−0.29	(−0.46 to −0.13)	**
Atracurium (mg/kg)	−0.30	(−0.65 to 0.05)	

Note

Effect sizes are presented as betas and should be interpreted as follows: an increase in one unit of the covariate will increase the blood pressure Z‐value (standardized pre‐incision non‐invasive blood pressure) by beta times the SD and 95% confidence intervals (CI).

* *P* value < .05.

** *P* value < .001.

## 7 DISCUSSION

### 7.1 Overall results

We were not able to identify a “typical” child or procedure prone to have a low pre‐incision blood pressure. Instead, the group of children with a low blood pressure was quite heterogeneous. We found several associations with pre‐incision blood pressure, of which the association with the use of a loco‐regional technique was the most profound.

At first glance, patient factors such as age, preoperative blood pressure, bronchial hyperreactivity, epilepsy, kidney disorders, and surgical specialty had the largest influence on pre‐incision blood pressure. In comparison, in the anesthetic management, only the use of loco‐regional anesthesia, the type of airway management and sevoflurane concentration were factors associated with blood pressure. It is likely though that other unmeasured factors are taken in account by the anesthesiologist, which we are unable to collect objectively in retrospective data, for example anesthesia dosing strategy based on clinical experience and intuition. These factors may have a larger influence on blood pressure than those included in this study.

### 7.2 Comparison with previous research

When we compare our findings with previous research, we should first emphasize that the definition of hypotension in previous studies was different. For example, Nafiu et al used reference values based on blood pressure in awake children, instead of reference values in children under anesthesia.^2^, ^10^ Others, for example Weber et al, defined low blood pressure as a drop of blood pressure relative to the pre‐operative blood pressure of the patients.^19^ The factors age and pre‐operative blood pressure which we found to be associated with pre‐incision blood pressure in this study have been reported previously.^10^, ^19^

If we compare the association of pre‐incision blood pressure with patient and anesthesia characteristics between infants and children older than 12 months, we did observe some differences. But we have to take into account that the number of infants was considerably smaller than the overall population (n = 3858; 20% of overall population) and that some of the factors hardly occurred in this age group, such as for example epilepsy. This has lowered the power to detect a potential association.

### 7.3 Strengths and limitations

To our knowledge, only a limited number of studies have tried to characterize children with a low pre‐incision blood pressure.^9^, ^11^, ^20^ The large sample size and broad inclusion criteria are strengths of the present study. We aimed to facilitate interpretation using gender and height adjusted references for normalizing mean blood pressure.

This study was designed to evaluate the reference values for blood pressure during anesthesia which we published previously. We aimed to gain more information about those children who are below the normal range. Therefore, we preferred to use the same data collection method to prevent bias, that is, to focus only on the pre‐incision period, using the last three measurements before incision. We did not explore other methodological options, such as analyzing the pattern of all or part of the blood pressure measurements or summarizing blood pressures in a hypotension metric, for example the minimum blood pressure or the area under a threshold. These methods quantify intraoperative hypotension, requiring a choice in method and, in most cases, a hypotension threshold, which is a source of discussion in hypotension research in adults.^21^, ^22^ Although hypotension quantification was not the aim of the present study, this could be interesting for future research. The current study can be viewed as an initial step in this direction because quantifying hypotension using a standardized blood pressure makes more sense than applying the same hypotension definition to neonates, children, and young adults.

When interpreting the findings of this study, we also need to acknowledge several limitations. First of all, this study was designed as a retrospective observational explorative study. Hence, the findings of this study cannot directly be generalized to clinical practice at this point but should be considered as hypothesis generating for further research. Second, the reference curves we used were developed on a multi‐center dataset, containing predominately US centers, including relatively healthy children (ASA physical status 1 and 2).^2^ Our study population was different to this population, since we also included higher ASA physical status. Consequently, potential misfit of the reference curves may have biased our findings. We have some indication that this phenomenon existed in the data, which we studied. We expected age not to be associated with blood pressure because we adjusted blood pressure indirectly for age when we calculated the Z‐values. Nevertheless, age remained associated after a multivariate model was fit. We also cannot rule out that other factors were associated with blood pressure, as a consequence of collinearity with age. Third, we did not take into account measurement aspects of pre‐incision blood pressure such as patient position, blood pressure cuff size used, or manipulation of the patient during measurement. These aspects were not reliably registered in our AIMS data. Not taking measurement aspects into account may lead to artifacts, extra variation or bias in the data.^23^

## 8 CONCLUSION

In conclusion, the population of children with a low pre‐incision blood pressure is heterogeneous and therefore we cannot describe a typical pediatric patient prone for low blood pressure during surgery. Although pre‐incision blood pressure is associated with choices in anesthesia technique, for example with loco‐regional anesthesia technique, we do not think that the data of the present study indicate that current clinical practice should be altered in favor of the intraoperative blood pressure. The data presented is a representation of a safe anesthesia practice, in which low blood pressure can occur and is also managed adequately.

## CONFLICT OF INTEREST

None of the authors have a conflict of interest regarding this research project.

## AUTHOR CONTRIBUTIONS

Mr W. Pasma designed and coordinated the study, performed data extraction, processing, performed and finalized statistical analysis, drafted the initial manuscript, and reviewed and revised the manuscript. Ms van den Broek substantially contributed to the concept and design of the study, carried out initial data analyses drafted the initial manuscript, substantially contributed to the interpretation of the results and critically reviewed and revised the manuscript. Dr Peelen and Dr de Graaff substantially contributed to the concept and design of the study, data acquisition, statistical analysis and interpretation of the results, and critically reviewed and revised the manuscript. Prof. van Buuren and Prof. van Klei substantially contributed to the concept and design of the study, statistical analysis and interpretation of the results, and critically reviewed and revised the manuscript. All authors approved the final manuscript as submitted and agree to be accountable for all aspects of the work.
