# Supplementary material for: Patient and anesthesia characteristics of children with low pre‐incision blood pressure: A retrospective observational study
Source: Acta Anaesthesiol Scand. 2019 Dec 22;64(4):472–80. doi: 10.1111/aas.13520 (PMC7079014; doi:10.1111/aas.13520)
Supplement: Supplementary file 1 [file AAS-64-472-s001.pdf]

Supporting data S1: Relevant period for height measurements

Age	Relevant period
0 to 1 months	7 days
1 to 3 months	14 days
6 to 12 months	30 days
1 to 4 years	60 days
>4 years	90 days

Height measurements were collected, within a relevant period before surgery. When there was no height measurement available within this period, height was considered missing.
